# Changes in the Gut and Oral Microbiome in Children with Phenylketonuria in the Context of Dietary Restrictions—A Preliminary Study

**DOI:** 10.3390/nu16223915

**Published:** 2024-11-16

**Authors:** Malgorzata Ostrowska, Karolina Nowosad, Bozena Mikoluc, Hubert Szczerba, Elwira Komon-Janczara

**Affiliations:** 1 Department of Biotechnology, Microbiology and Human Nutrition, University of Life Sciences in Lublin, 8 Skromna St., 20-704 Lublin, Poland; malgorzata.ostrowska@up.lublin.pl (M.O.);; 2 Department of Pediatrics, Rheumatology, Immunology and Metabolic Bone Diseases, Medical University of Bialystok, 15-274 Bialystok, Poland

**Keywords:** phenylketonuria (PKU), 16S rRNA, gut microbiome, oral microbiome, diet, QIIME2

## Abstract

Background: Phenylketonuria (PKU) is a metabolic disorder that necessitates dietary restrictions, potentially impacting the composition of gut and oral microbiota. This study aimed to compare the microbiota composition between children with PKU and healthy controls. Methods: Using 16S rRNA gene sequencing, we analysed microbial communities at six phylogenetic levels. Results: Our findings revealed significant differences in the gut microbiota: Euryarchaeota was more abundant in controls (*p* = 0.01), while Bacilli and Lactobacillales were higher in PKU children (*p* = 0.019). Methanobacteriales were significantly elevated in controls (*p* = 0.01). At the genus and species levels, PKU children had higher Streptococcus and *Eubacterium dolichum* (*p* = 0.019, *p* = 0.015), whereas controls had more *Barnesiella*, *Coprococcus*, and *Faecalibacterium prausnitzii* (*p* = 0.014, *p* = 0.019, *p* = 0.014). In the oral microbiota, control children exhibited significantly higher Bacteroidetes (*p* = 0.032), while PKU children had increased Bacilli and Betaproteobacteria (*p* = 0.0079, *p* = 0.016). *Streptococcus* and *Neisseria* were more prevalent in PKU (*p* = 0.0079, *p* = 0.016). Conclusions: These results suggest that PKU and its dietary management significantly alter the gut and oral microbiota composition. Understanding these microbial shifts could have implications for managing PKU and improving patient outcomes.

## 1. Introduction

Phenylketonuria (PKU, OMIM 261600) is an autosomal recessive inherited disease characterised by elevated plasma phenylalanine (Phe) levels due to mutations in the phenylalanine hydroxylase (PAH). PAH (EC 1.14.16.1) catalyses the hepatic conversion of Phe to tyrosine (Tyr), with tetrahydrobiopterin (BH4) as a cofactor. A global frequency of 1:23,930 live births (range of 1:4500 [Italy], 1:8309 [Poland], 1:15,924 [China], and 1:125,000 [Japan]) was estimated for 0.45 million PKU cases in 2020 [1]. Since May 1994, every neonate born in Poland has had to take tests to check for a diagnosis of PKU [2]. The treatment of this disease consists of limiting Phe intake, supplementation with essential amino acids and microelements, and the administration of large neutral amino acids (LNAAs) or glycomacropeptides (GMPs) [3]. The majority of neurological disorders can be prevented with early dietary intervention [4]. Recent studies have examined oral dosing of SYNB1618 and SYNB1934 strains with a higher Phe ammonia lyase activity of modified *Escherichia coli* Nissle 1917 to metabolise Phe in the gut. Postprandial and fasting plasma Phe levels in PKU patients can be lowered using gut-metabolised synthetic biotics [5]. Untreated patients usually develop mental retardation, seizures, or other neurological symptoms. According to European PKU Phe tolerance guidelines, blood Phe levels should be 120–360 μmol/L for children under 12 and women on a preconception or pregnancy diet and 120–600 μmol/L for patients over 12 [6]. Phenylketonuria in newborns needs to be diagnosed quickly, treated with a Phe-free diet, and maintained throughout life. It can be difficult for patients to adhere to a restrictive diet, so it is essential to seek alternative treatment options.

A comparative analysis was conducted to research the gut microbiota of individuals with phenylketonuria (PKU) who followed a diet low in Phe in comparison to healthy children from several countries, including Brazil [7], Italy [8], and China [9]. The study revealed variations in the microbiome across the groups being studied in the respective nations. The researchers proposed that plasma Phe levels may cause disparities in the microbiota. The PKU group had a lower abundance of microorganisms involved in starch and carbohydrate metabolism; glycolysis/gluconeogenesis; and Phe, Tyr, and tryptophan (Trp) production compared to the control group [7]. Altering the dietary treatment of individuals with PKU to decrease their consumption of Phe may lead to an imbalance distribution of Firmicutes and Bacteroidetes bacteria, which has been linked to higher body mass index (BMI) and obesity in adolescents. The microbiota of PKU patients may be affected due to a persistent reduction in amino acid (AA) consumption, which is frequently not effectively replaced by protein substitutions. This alteration may lead to changes in microbial composition, less efficient management of glucose levels, increased insulin resistance, and weight gain [10].

The oral microbiome has also been studied in association with human diseases, in addition to the gut microbiome. Oral bacteria are capable of colonising the gut, and changes in the oral microbiome are associated with various diseases, such as caries [11], type II diabetes [12], and obesity [13]. The presence of some species is associated with diet, e.g., following a ketogenic diet results in a decrease in the relative number of *Haemophilus*, *Neisseria*, and *Prevotella* [14]. Understanding the oral microbiome and its relationships with other microbiomes and health statuses is essential to improving human health. Changes in the saliva microbiota may signal host systemic diseases. Additionally, oral hygiene and food can affect the oral microbiome [15]. In older children and adults, elevated Phe levels compound the difficulty of maintaining a restrictive diet. Recent research suggests that defective Trp metabolism and gut microbiota alterations may affect a wide range of gut–brain axis (GBA) disorders. Childhood diets can alter brain development and adult cognition. Patients with phenylketonuria treated with Phe-free amino acid medical food (AA-MF) have altered Trp metabolism and an imbalanced kynurenine (KYN) pathway, resulting in the formation of neurotoxic metabolites involved in many neurodegenerative and inflammatory disorders [16].

The object of this study was to identify differences between the oral microbiome and the gut microbiome in PKU children compared to a healthy control group. A dietary analysis was also performed to show differences between the study cases. The use of a special phenylalanine-free diet in PKU patients, as well as the patient’s age, can impact the biodiversity of the oral or gut microbiome. The diet and genetic predisposition, together with the living environment (including geographic location), might generate significant differences in the microbiome of patients with PKU compared to controls in the Polish population.

## 2. Materials and Methods

### 2.1. Patients and Cohorts

The preliminary study was carried out on 10 children from the Polish population. They were divided into two study groups of 5 patients each: a group diagnosed with PKU and a control group unrelated to PKU. The groups were selected based on ‘pair matching’, i.e., according to individual concordance in the form of setting the control group with the test group in pairs compatible in terms of matching variables, e.g., age. Individual matching was intended to indicate differences in health status and diet. The Dietary Clinic of the University of Life Sciences in Lublin and other metabolic disease centres in Poland recruited participants for both cohorts of the study. The inclusion criteria for the PKU study group included a diagnosis of PKU during newborn screening, an age of less than 18 years, and a current Phe level test. The control group’s inclusion criteria included no association with PKU and an age of less than 18 years. Exclusion criteria for both groups included congenital malformations, chronic liver disease, chronic or acute intestinal disease, antibiotic administration, and use of probiotics within 3 months before study entry.

Each person was given a dietary questionnaire with questions on age, gender, height, weight, past medical history, medications used, lifestyle, food diary (3 days), and frequency of consumption of different food groups. Parents and legal guardians were given instructions on how to weigh and record meals. All data were fully anonymised prior to access by the authors.

### 2.2. Sample Collection

The collection of faecal and salivary biological material took place at the same time as the collection of dietary data. Biological material was collected into appropriately labelled storage tubes. The biological material of saliva was collected into special GeneFix™ Saliva Microbiome DNA Collector tubes (Isohelix™, Kent, UK) and stored at −20 °C until analysis. Stool samples were collected using the Stool Sample Collection & Stabilization method (Canvax, Valladolid, Spain) and stored at −20 °C.

### 2.3. Microbial Sequencing—16S rRNA

Oral microbiome DNA was isolated using GeneFiX™ DNA Isolation Kits (Isohelix™, Kent, UK) according to the procedure. In contrast, the DNA of the microbiome from the stools was isolated using the HigherPurity™ Stool DNA Isolation Kit (Canvax, Valladolid, Spain) and stored at −20 °C until molecular analysis. The concentration of the material was then determined using the Qubit 4.0 fluorometer (Invitrogen, Waltham, MA, USA) and the purity on NanoDrop™ 2000 (Thermo Fisher Scientific, Waltham, MA, USA). Only DNA samples with purity ratios 260/280 > 1.8 and 260/230 > 1.8 and concentration ≥5 ng/µL were used in the study. For each 25 ng DNA sample, PCR was performed with 2 × KAPA HiFi HotStart ReadyMix polymerase (Roche Kapa Biosystems, Wilmington, NC, USA) for the specific hypervariable region of bacterial 16S rRNA (V4) using following primers: 515F [17] and 806R [18] (with adapter sequences: forward 5′ TCGTCGGCAGCGTCAGATGTGTATAAGAGACAG-[locus-specific sequence] and reverse 5′ GTCTCGTGGGCTCGGAGATGTGTATAAGAGACAG-[locus-specific sequence]), as described in the 16S Metagenomic Sequencing Library Preparation Protocol (Illumina, San Diego, CA, USA). The following thermal cycling parameters were used for the amplification of DNA: 95 °C for 3 min and 25 cycles of 95 °C for 30 s, 55 °C for 30 s, 72 °C for 30 s, and a final extension at 72 °C for 5 min. DNA amplicons were purified using Agencourt^®^ AMPure^®^ XP magnetic beads (Beckman Coulter, Brea, CA, USA) following the manufacturer’s instructions. Next, the libraries were double-indexed using the Nextera XT kit according to the manufacturer’s instructions (Illumina). PCR of 50 μL was performed under the following conditions: 95 °C for 3 min and 8 cycles of 95 °C for 30 s, 55 °C for 30 s, 72 °C for 30 s, and a final extension at 72 °C for 5 min. The final libraries were cleaned up using Agencourt^®^ AMPure^®^ XP magnetic beads (Beckman Coulter, Brea, CA, USA). Libraries were quantified using Qubit dsDNA BR assay (Invitrogen, Waltham, MA, USA). The size of the libraries’ 420–450 base pairs (bp) was checked using TapeStation Desktop Systems (Agilent Technologies, Santa Clara, CA, USA). Then, libraries were pooled at equimolar concentrations, ensuring normalisation across the different samples sequenced in the same run. The final concentration was 50 pM. All libraries were sequenced with the Illumina PE 2 × 150 using iSeq 100 i1 Reagent v2 Kit (300 cycles) (Illumina Inc., San Diego, CA, USA) with the iSeq 100 Instrument (Illumina Inc., San Diego, CA, USA). System protocol was modelled according to the specifications of the manufacturer. As an internal control for a low-diversity library, 10% of PhiX viral DNA was added to the sample pool. 16S sequencing raw data were submitted to the NCBI SRA gut microbiome under the accession number PRJNA1161376 and oral microbiome accession number PRJNA1161359.

### 2.4. Microbiome Data Processing

Bioinformatic analyses on gut and oral microbiota 16S rRNA were conducted using the QIIME 2 pipeline 2020.2 [19]. After sequencing, the raw sequence data (FASTQ) were imported into QIIME 2. In the next step, data were demultiplexed, quality was filtered using the q2-demux plugin, and sequence denoising was carried out using DADA2 [19]. Quality filtering, length trimming, and clustering filters were read into the operational taxonomic unit (OTU) at 99% identity level and discarding singletons as possible chimaeras. Alpha diversity was computed through the QIIME2 pipeline using the Chao1; the number of OTUs; Shannon diversity, which measures the entropy of classification at the species level in the samples; and Faith’s Phylogenetic Diversity whole tree (PD whole tree) metrics. To compare the microbial community structure of the subjects, weighted and unweighted UniFrac distances, Jaccard distance, and Bray–Curtis dissimilarity were used. Scatter plots were used to display Principal Coordinate Analysis (PCoA) results for the normalised relative abundance of all samples. PERMANOVA and ANCOM evaluated sample differences by abundance or distribution. The taxonomic analysis was performed via the OTUs classifier against the Greengenes2 [release 2022.10.backbone.full-length.fna.qza [20] from phylum to species level. We used ALDEx2 (version 1.34.0) a differential .abundance package, to investigate the differences in gut and oral abundance in two groups [21].

### 2.5. Statistical Analysis

The non-parametric Wilcoxon Rank-Sum Test on RStudio http://www.rstudio.com/, (accessed on 29 January 2024) (R version 4.3.1 Foundation for Statistical Computing, Vienna, Austria; used by RStudio, Inc., Boston, MA, USA) [22] was used to find differences in clinical, nutritional, and taxonomic data between match samples. Next, the qiime2R package (version 0.99.6) [23] was used to import and analyse microbiome data, which allowed the integration of QIIME2 artefacts into the R workflow and facilitated the downstream analysis of microbiome sequencing data within the R environment. We used functions from the microbiome R package (version 1.24.0) [24] and vegan (version 2.6-4) [25] to conduct diversity analyses (alpha and beta diversity and PERMANOVA) and differential abundance tests after data preprocessing. Various taxa summaries and relative abundances were computed and visualised. This package provides functionality for data wrangling, community analysis, and visualisation of microbiome taxon data. We enhanced our microbiome analysis with the microbiome utilities package (version 1.00.17) [26], which provides tools for managing and visualising microbiome data, specially designed for the creation of publication-ready plots and emphasising essential features such as taxonomic composition and statistical comparisons between sample groups. We used DECIPHER (version 2.30.0) [27] to manage extensive biological sequencing data, including importing, maintaining, analysing, modifying, and exporting. Software DESeq2 (version 1.42.1) [28], in conjunction with phyloseq (version 1.46.0) [29], was used for differential expression analysis of the microbiota. Phyloseq offered data management and visualisation functionalities for microbiome taxon data. We created taxonomic bar plots and diversity indices to visually represent community compositions, used ggplot2 (version 3.4.4) [30] with RColorBrewer (version 1.1-3) [31] for colour palettes, and used ggrepel (version 0.9.5) [32] to prevent overlapping text labels. This package allows for creating complex multi-layered graphics with an intuitive syntax following the Grammar of Graphics principles. The R package ‘ggplots2’ will assess taxonomic correlations and other food diary data using an asymptotic *p*-value method. All *p*-values < 0.05 were considered significant.

Kcalmar.com https://kcalmar.com/, (accessed on 15 February 2024) was used to analyse the daily nutritional value. Data from the food diaries were analysed according to European Food Safety Authority (EFSA) standards [33]. The results collected from the food diaries were statistically analysed using Statistica 13.3. The level of significance was *p* > 0.05. In order to check the existence of statistical significance between groups, Student’s *t*-test for two independent samples (for normal distribution) and Mann–Whitney U test (if the distribution was not normal) were used. An analysis of the False Discovery Rate (Benjamini–Hochberg) was conducted to avoid Type I errors. The result was considered statistically significant if the *p*-value < 0.05.

## 3. Results

### 3.1. Cohort Description

This study involved 10 children (5 in the PKU group and 5 in the control group). In the PKU group, 60% were girls and 40% were boys, whereas in the control group, 80% were girls and 20% were boys. The average age in the PKU group was 5.85 years with a standard deviation (SD) of 5.13, while in the control group, it was 7.25 years with an SD of 3.86. Both groups had a similar average body weight, with the PKU group weighing 29.65 kg (SD 23.49) and the control group weighing 29.58 kg (SD 16.36). The PKU group had a mean height of 117.25 cm (SD 31.95), and the control group’s was 125.75 cm (SD 23.89).

Most parents of the children studied noted the presence of animals at home. Also, in the case of the PKU group, the parents were asked to enter the latest results of Phe (the mean in the PKU group was 5.40 ± 2.90 mg/dL) and Tyr levels (the mean in the PKU group was 1.37 ± 0.78 mg/dL). Their daily protein intake was largely replaced by PKU2 (Milupa, Friedrichsdorf, Germany), which was a Phe-free mixture of amino acids.

### 3.2. Nutritional Assessment

Comparing the nutrition of children with phenylketonuria with healthy children is crucial to understanding the differences in their dietary needs and the impact of diet on their development. The average energy intake in the group of patients with phenylketonuria was higher than in the control group and amounted to 1684 ± 748 kcal (Table 1). The most common products in the diet of these people are fruits, some vegetables, and oils. People with PKU also consume products with a reduced Phe content, e.g., bread, pasta, rice, or cereals, which was also observed when analysing the diet of patients (significant differences in Phe intake between groups). Significant differences between groups were also observed in the case of plant protein consumption. The average total protein consumption in the PKU group was 41.28 ± 5.83 g, while in the control group, it was 61.43 ± 17.88 g.

In the case of minerals, no significant differences were observed between groups. A statistically significant difference was observed for folates (Table 1). Differences in the intake of vitamin B12 and vitamin D were noted between groups; however, these differences were not statistically significant (Table 1).

### 3.3. Gut Microbiota Composition in PKU and Control Children

**Figure 1:**
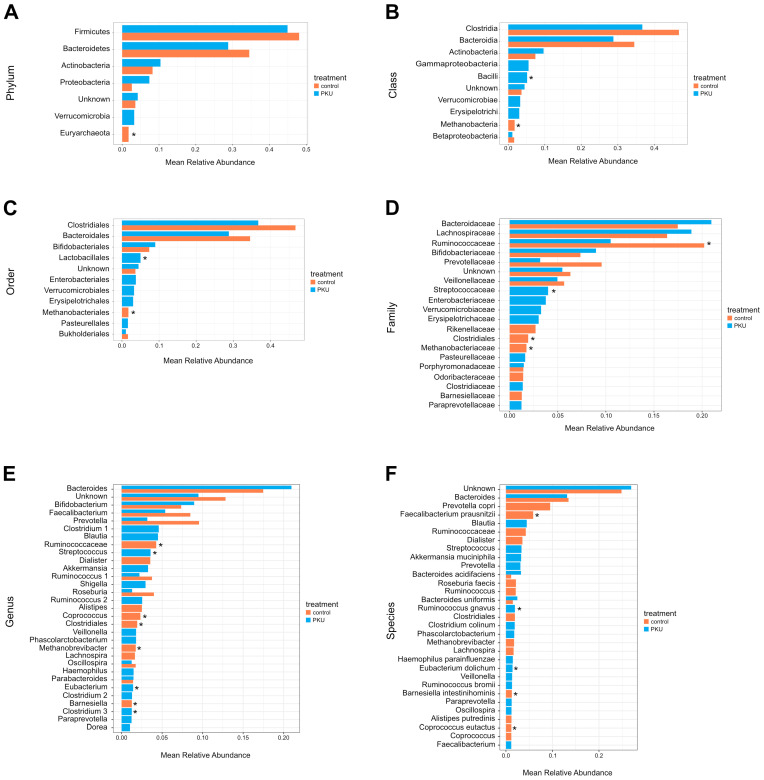
(**A**–**F**) Comparison of gut microbiota composition between PKU and healthy control children. The bar plots represent the mean relative abundance of the six taxonomic levels: (**A**) phylum, (**B**) class, (**C**) order, (**D**) family, (**E**) genus, and (**F**) species (>0.01 mean relative abundance). The asterisk indicates a significant correlation with a * *p*-value < 0.05.

The gut microbiota composition from the phylum to the species is depicted in Figure 1A–F, and the relative abundances at six phylogenetic levels are reported in Supplementary Tables S1–S6 (based on a >1% abundance). The most relatively abundant phyla in PKU and control group were Firmicutes (44.90% for PKU vs. 48.05% for the control, *p* = 0.71) and Bacteroidetes (28.79% for PKU vs. 34.51% for the control, *p* = 0.71). The Firmicutes/Bacteroidetes ratio was (PKU = 1.55 vs. control = 1.39). Of all phyla, only Euryarchaeota (1.75% for the control, *p* = 0.01) had statistically significant differences in their abundance from the control (Figure 1A, Table S1). The bacterial classes Bacilli (5.17% for PKU vs. 0.90% for the control, *p* = 0.019) showed higher mean relative abundance in PKU (Figure 1B, Table S2). The abundance of Lactobacillales (5.01% for PKU vs. 0.77% for the control, *p* = 0.019) was significantly lower in the control group compared to the PKU. On the other hand, Methanobacteriales (1.75% for the control, *p* = 0.01) were more relatively abundant in the control, with data being significant (Figure 1C, Table S3). In contrast, control children were characterised by a significant increase in the relative abundance of *Ruminococcaceae* (10.50% for PKU vs. 20.22% for the control, *p* = 0.019) and *Methanobacteriaceae* (1.75% for the control, *p* = 0.01). Furthermore, we observed an increased *Streptococcaceae* (4.0% for PKU vs. 0.77% for the control, *p* = 0.019) in PKU compared to control children (Figure 1D, Table S4). Children with PKU were characterised by an increase in the relative abundance of *Streptococcus* (3.58% for PKU vs. 0.77% for the control, *p* = 0.019) and *Eubacterium* (1.42% for PKU, *p* = 0.015), statistically significant. In contrast, *Barnesiella* (0.21% for PKU vs. 1.26% for the control, *p* = 0.014), *Coprococcus* (0.75% for PKU vs. 2.32% for the control, *p* = 0.019), and *Methanobrevibacter* (1.75% for the control, *p* = 0.01), were more relatively abundant in control children (Figure 1E, Table S5). Similar levels of abundance were observed for PKU children for *Eubacterium dolichum* (1.42% for PKU, *p* = 0.015) and *Ruminococcus gnavus* (1.93% for PKU, *p* = 0.044). In contrast, control children were characterised by a significant increase in the relative abundance of *Barnesiella intestinihominis* (0.21% for PKU vs. 1.26% for the control, *p* = 0.014), *Coprococcus eutactus* (1.17% for the control, *p* = 0.01), and *Faecalibacterium prausnitzii* (0.53% for PKU vs. 5.86% for the control, *p* = 0.014) (Figure 1F, Table S6).

**Figure 2:**
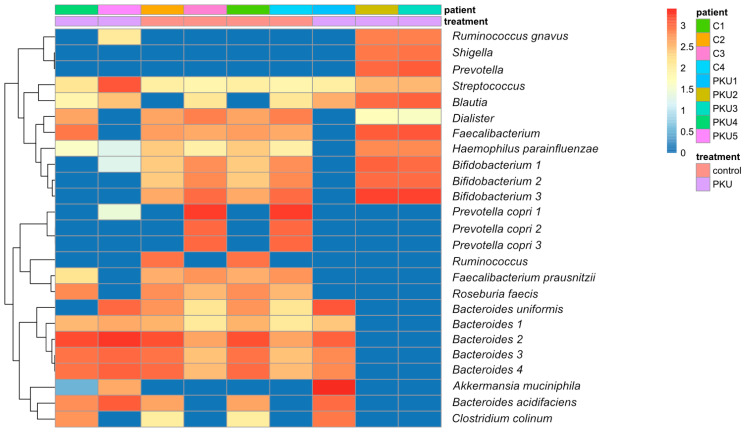
Heatmap of gut microbiota abundance in the control and PKU groups. Group of children with phenylketonuria (PKU1, PKU2, PKU3, PKU4, and PKU5) compared to the control children (C1, C2, C3, and C4) group. Each row represents a different bacterial genus or species. The colour gradient indicates levels of abundance, with dark blue indicating very little or no presence and red indicating the most abundant. The colour bar above the heatmap shows the treatment status for control and PKU children.

A heatmap was used to display a comparison of the intestinal microbiota composition between children with PKU and healthy controls (Figure 2). Some PKU patients (PKU2 and PKU3) have a greater prevalence of taxa such as *Ruminococcus gnavus*, *Shigella*, and *Prevotella* compared to the control group. However, certain taxa, such as *Bifidobacterium* (C1–C4, PKU2–PKU3), *Bacteroides* (C1–C4, PKU1, PKU4, PKU5), *Blautia* (PKU1–PKU5, C3, C4), *Faecalibacterium* (PKU2-PKU4, C1–C4), *Faecalibacterium prausnitzii* (PKU4; C1–C4), *Prevotella copri* (C3–C4), *Rumminococcus* (C1, C2), and *Roseburia faecies* (PKU1, C1–C4), indicated varying levels of abundance in the two groups. The dendrogram shown on the left side of the heatmap illustrates the clustering of genera according to their patterns of abundance.

Next, analyses were conducted to determine the overall abundance of core members in each sample by summing their abundances in PKU and control. The heatmap displayed the detection thresholds, represented as relative abundance, of several microbial taxa in the intestinal samples obtained from individuals with PKU (Figure S1). *Streptococcus*, *Blautia*, *Lachnospiraceae*, *Oscillospora*, *Eggerthella lenta*, and *Faecalibacterium* were found to have high frequencies in the samples from PKU.

**Figure 3:**
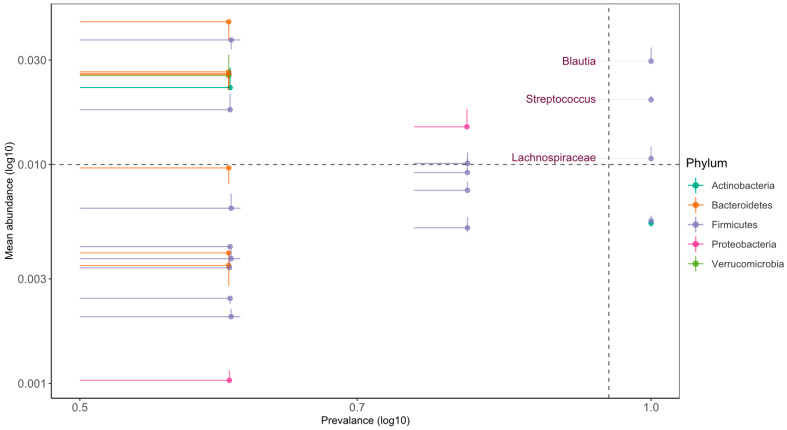
Gut microbiome bacterial taxonomic predominance and mean abundance in PKU. The scatterplot showed the relationship between the prevalence and mean abundance of various bacterial taxa in the gut microbiome, with both axes on a logarithmic scale. The *x*-axis represents the prevalence (log10), and the *y*-axis represents the mean abundance (log10). Each point represents a different bacterial taxon, colour-coded by phylum. The error bars indicate variability in abundance and prevalence. The dashed line represents a threshold separating taxa with a mean abundance higher than 0.010 log 10.

Taxa related to the Firmicutes phylum (*Blautia* and *Lachnospiraceae* families) and from the Proteobacteria phylum (*Streptococcus*) tended to be prevalent and highly abundant in the PKU gut microbiome (Figure 3). These taxa had a prevalence of over 1.0 log 10 and a mean abundance of more than 0.010 log 10. The phyla Bacteroidetes and Verrucomicrobia exhibited a range of prevalence and abundance, indicating that these groups have a wide-ranging presence in the gut microbiome. Taxa that were present in the upper right quadrant, characterised by high prevalence and high abundance, may be classified as components of the core microbiome. In contrast, taxa detected in the lower left quadrant, characterised by low frequency and low abundance, may represent transient or less dominant taxon (Figure 3).

Several taxonomic groups, including *Bacteroides*, *Faecalibacterium*, *Bifidobacterium*, *Ruminococcaceae,* and *Prevotella,* were widely distributed, highly prevalent, and relatively abundant, indicating that they played a crucial role in the essential gut microbiome in the control.

### 3.4. Oral Microbiota Composition in PKU and Control

**Figure 4:**
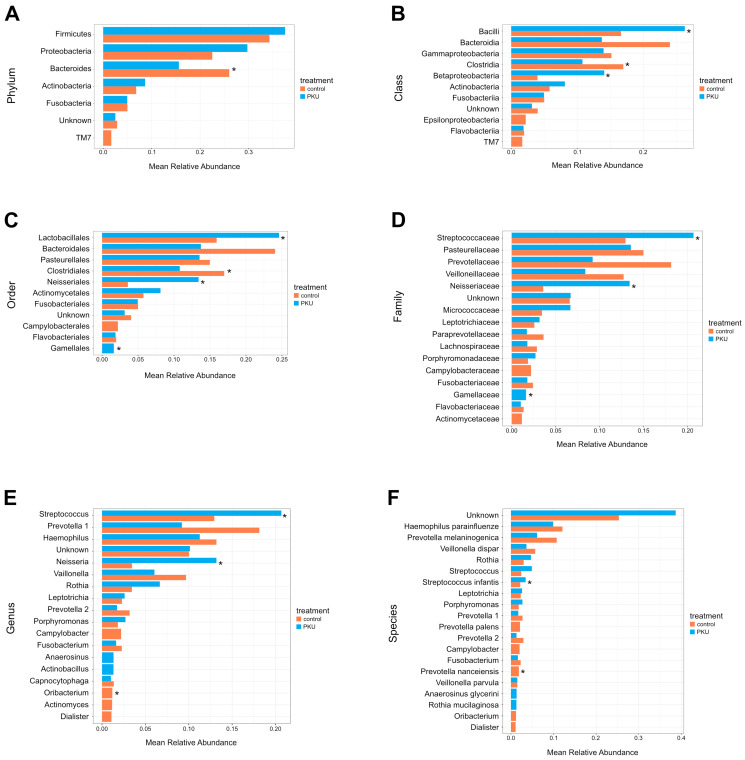
(**A**–**F**) Comparison of oral microbiota composition between PKU and healthy control children. The bar plots represent the mean relative abundance of the six taxonomic levels: (**A**) phylum, (**B**) class, (**C**) order, (**D**) family, (**E**) genus, and (**F**) species (>0.01 mean relative abundance). The asterisk indicates significant correlation. * indicates *p*-value < 0.05.

The oral microbiota composition from the phylum to the species is depicted in Figure 4A–F, and the relative abundances at six phylogenetic levels are reported in Supplementary Tables S8–S13 (based on a >1% abundance). The most relatively abundant phyla in the PKU and control groups were Firmicutes (37.52% for PKU vs. 34.29% for the control, *p* = 0.22). The control group (26.00%) had a significantly higher abundance of Bacteroidetes compared to the PKU (15.61%) (*p* = 0.032) (Figure 4A, Table S8). Significant differences were observed in the abundances of PKU microbiota Bacilli (26.30% for PKU vs. 16.64% for the control, *p* = 0.0079) and Betaproteobacteria (14.10% for PKU vs. 3.98% for the control, *p* = 0.016). On the other hand, Clostridia (10.80% for PKU vs. 16.98% for the control, *p* = 0.032) was more relatively abundant in control children (Figure 4B, Table S9). Lactobacillales was the predominant order in PKU (24.60% for PKU vs. 15.92% for the control, *p* = 0.0079). Furthermore, the abundance of Neisseriales (13.41% for PKU vs. 3.59%, *p* = 0.016) and Gemellales (1.64% for PKU vs. 0.71% for the control, *p* = 0.032) was significantly lower in the control group compared to the PKU. In contrast to this, Clostridiales (10.80% for PKU vs. 16.98% for the control, *p* = 0.032) was more relatively abundant in the control (Figure 4C, Table S10). The abundance of *Streptococcaceae* (20.68% for PKU vs. 12.94% for the control, *p* = 0.0079) was significantly higher in the PKU. *Neisseriaceae* (13.41% for PKU vs. 3.59% for the control, *p* = 0.016) and *Gemellaceae* (1.64% for PKU vs. 0.71% for the control, *p* = 0.032) were significantly higher in the PKU (Figure 4D, Table S11). *Streptococcus* (20.68% for PKU vs. 12.94% for the control, *p* = 0.0079) and *Neisseria* (13.19% for PKU vs. 3.46% for the control, *p* = 0.016) were the predominant genera in PKU compared to the control, with data being significant. In contrast, *Oribacterium* (0.75% for PKU vs. 1.19% for the control, *p* = 0.016) showed a decreasing trend in children with PKU. (Figure 4E, Table S12). *Streptococcus infantis* (3.45% for PKU vs. 2.19% for the control, *p* = 0.032) indicated a higher prevalence in the PKU group; on the other hand, *Prevotella nanceiensis* (0.50% for PKU vs. 1.86% for the control, *p* = 0.032) was higher in the control, and data were statistically significant (Figure 4F, Table S13).

**Figure 5:**
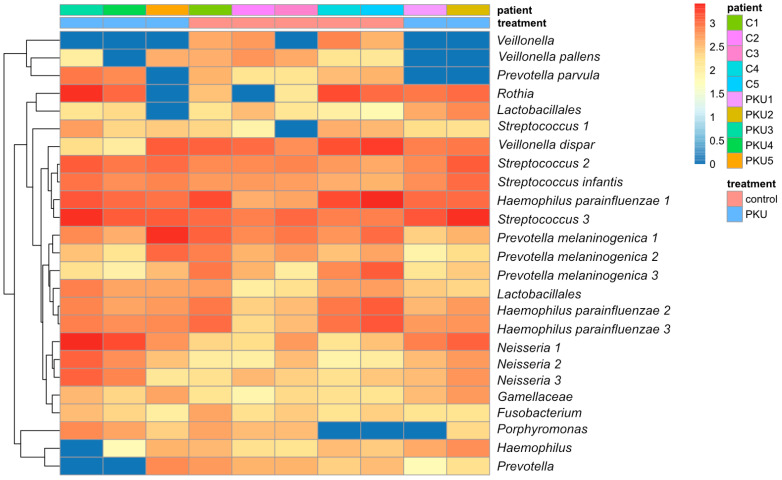
Heatmap of oral microbiota abundance in the control and PKU groups. Group of children with phenylketonuria (PKU1, PKU2, PKU3, PKU4, and PKU5) compared to the group of control children (C1, C2, C3, C4, and C5). Each row represents a different bacterial genus or species, identified by unique codes followed by names. The colour gradient indicates levels of abundance, with dark blue indicating very little or no presence and red indicating the most abundant. The colour bar above the heat map shows the treatment status for control and PKU children.

The oral microbiota composed of PKU children and healthy controls was compared using a heatmap (Figure 5). Streptococcus and Neisseria were found to be present in substantial amounts in PKU patients and controls. *Veillonella* exhibited significantly higher levels of abundance in the control patients (C1, C2, C4, and C5) as compared to the PKU patients. *P. melaninogenica* and *P. pallens* (PKU1, PKU2, and PKU4) showed modest variation in abundance in PKU and the control or no presence at all (shown in brackets). In addition, *Rothia* demonstrated greater prevalence in both the control group (C4) and PKU (PKU3) patients and was absent in C2 and PKU5. *Porphyromonas* was identified by its low abundance, being minimally present in most samples or deficient in prevalence in C4, C5, and PKU1.

The analysis of taxon frequency in the PKU group revealed that some bacterial taxa, including *Streptococcus*, *S. infantis*, *Rothia mucilaginosa*, *Neisseria*, and *Gemellaceae*, demonstrated a high prevalence and relative abundance. *Veillonella* and *Actinomyces* were taxa that had a moderate frequency of occurrence. Nevertheless, taxa such as *Campylobacter* and *Treponema* were less prevalent, occurring in a smaller amount of samples and often with lower levels of detection (Figure S2).

**Figure 6:**
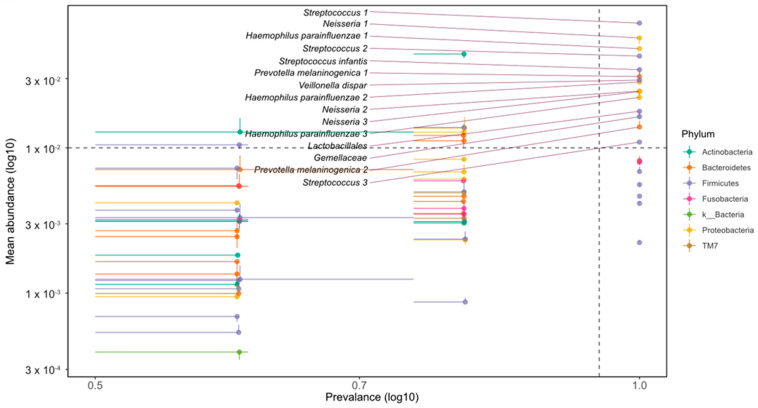
Oral microbiome bacterial taxonomic predominance and mean abundance in PKU. The scatterplot shows the relationship between the prevalence and mean abundance of various bacterial taxa in the oral microbiome, with both axes on a logarithmic scale. The *x*-axis represents the prevalence (log10), and the *y*-axis represents the mean abundance (log10). Each point represents a different bacterial taxon, colour-coded by phylum. The error bars indicate variability in abundance and prevalence. The dashed line at 10^−2^ x log(10) represents a threshold separating taxa with a higher mean abundance, considered potentially more biologically significant.

We analysed the frequency and mean abundance of bacterial taxa in oral samples from people with PKU for comparative purposes (Figure 6). Several taxonomic groups of the genus *Streptococcus* showed a high occurrence and relatively high mean abundance compared to other groups. Taxonomic groups such as *Neisseria*, *Lactobacillales*, and *Gemellaceae* had a high prevalence but had different average abundances. Certain taxa with lesser prevalence, such as those belonging to the genera *Actinobacteria* and *Saccharibacteria* TM7, had a lower mean abundance.

### 3.5. Gut and Oral Microbiome Composition

#### Alpha and Beta Diversity Analysis of Microbial Composition

**Figure 7:**
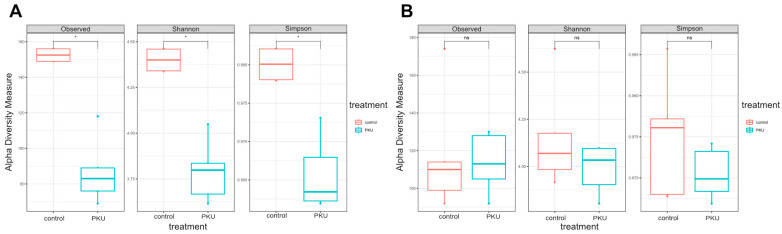
(**A**,**B**) Alpha diversity of taxa was identified in the gut microbiota between the control group and individuals with PKU (**A**), as well as in the oral microbiota between the control group and those with PKU (**B**). Alpha diversity analysis was conducted using many metrics to evaluate the variety within each sample and compare the number of species across different conditions being studied. Observed, Shannon, and Simpson diversity indices were estimated. The Wilcoxon rank-sum test was used to identify statistically significant differences. (*) *p* < 0.05 indicates a significant difference, whereas (ns) *p* < 0.05 indicates no significant difference.

To test the overall differences of microbial community structures in phenylketonuria children and controls (oral and gut), alpha diversity was measured using Observed, Shannon and Simpson indices. All data were statistically significant (*p* < 0.05) (Figure 7A, Table S7). The control group in the gut showed a higher median number of observed species compared to the PKU group. The control group had a higher Shannon index compared to the PKU group. This suggested that the control group not only had more species but also a more even distribution of species. The control group’s Simpson index was slightly higher than that of the PKU group. There were no significant variations in microbial diversity when comparing the alpha diversity measurements between the control and PKU treatment groups using oral samples (Figure 7B, Table S14).

To compare the microbial composition in PKU patients and controls, we used the Bray–Curtis (non-phylogenetic) and weighted Unifrac (phylogenetic) distance measures to estimate beta diversity. The PCoA ordination method was used for this analysis, and the results are demonstrated in Table S7. The gut microbial community composition in PKU samples showed more variety compared to control samples, which were more clustered. The Weighted UniFrac analysis revealed significant differences in the composition of microbial communities inside the intestine between the PKU and control groups (Table S7).

The analysis of both Bray–Curtis and weighted UniFrac PCoA scores reveals that the microbial communities in PKU patients were distinct from those in the control group in oral samples. Patients with phenylketonuria exhibit a more constrained and lower beta diversity in both Bray–Curtis and Weighted UniFrac metrics, suggesting that the oral microbiome in PKU individuals might be less diverse and more uniform than in control individuals. The control patients had more variability, particularly in Bray–Curtis ‘Axis 2’ and weighted UniFrac ‘Axis 2’ (Table S14).

### 3.6. PERMANOVA and Dispersion Analysis

The PERMANOVA analysis was conducted using the Bray–Curtis dissimilarity method with 99 permutations in samples from the gut samples. The treatment term had an R-squared value of 0.28059, an F-statistic of 2.7302, and a *p*-value of 0.05, which indicated statistical significance (Table S7). The Permutation Test for Homogeneity of Multivariate Dispersions investigated if the variability of the microbial communities is uniform across different groups. The study revealed that the groups had a degree of freedom (Df) of 1, a sum of squares (Sum_Sq) of 0.082371, and a mean square (Mean_Sq) of 0.082371. The calculated F-value of 126.88 based on 999 permutations and the *p*-value of 0.001 suggested a very significant difference in dispersion across the groups. The calculated *p*-value for the comparison between the control and PKU groups was 9.71 × 10^−6^, and the permuted *p*-value was 0.01. The PERMANOVA analysis revealed that the treatment had a statistically significant influence on the composition of the gut microbial community, accounting for about 28.06% of the observed variance (Table S7). PERMANOVA analysis was also performed to compare the oral microbiome of the control and PKU groups (Table S14). The treatment, specifically the difference between the control group and the group with PKU, explained approximately 24.4% of the overall difference in the organisation of the microbial community. The F-statistic of 2.5859, together with a *p*-value of 0.04, demonstrated that there was a statistically significant difference in microbial communities between the control and PKU groups. The high *p*-value (0.747) suggested that there was no significant difference in the dispersion (variance) between the control and PKU groups. Therefore, the notable outcome of the PERMANOVA analysis was most likely attributed to variations in the central tendency (location) of the community composition rather than variations in dispersion. The pairwise comparison revealed insignificant disparities between the control and PKU groups, as shown by high *p*-values.

### 3.7. Analysis of Differential Bacterial Abundance in PKU and Controls

**Figure 8:**
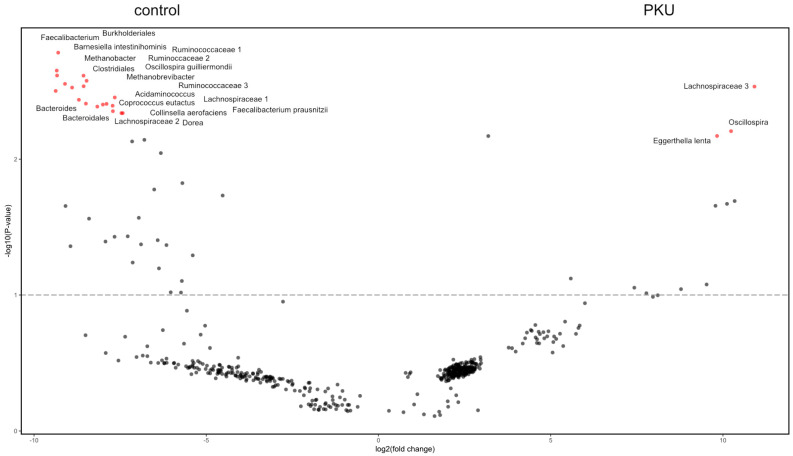
Differential taxon abundance analysis between gut PKU and control samples. The volcano plot illustrates the results of a differential microbial composition analysis comparing gut samples from the PKU group to the control group using the ALDEx2 method. The *x*-axis represents the log2 fold change in microbiota compositions between the two groups, with positive values indicating higher expression in the PKU group and negative values indicating higher expression in the control group. The *y*-axis represents the -log10 transformed *p*-values, with higher values indicating greater statistical significance. Each point on the plot corresponds to a single taxon. The red dots represent taxa that reached statistical significance with a higher level of confidence, often implying that these points have surpassed a certain threshold for significance. The dark dots indicate taxa that did not show statistical significance. The dashed line marks the significance threshold at *p* = 0.1 (-log10(0.1)).

The volcano plot was generated by analysing the differential abundance of compositions in the gut using ALDEx2. The negative numbers on the left side represent traits that were less prevalent in PKU, like *Ruminococcaceae*, *Coprococcus*, *Methanobrevibacter*, *Dorea*, *Barnesiella*, *Lachnospiraceae*, *Acidaminococcus,* and *Oscillospira,* compared to the control. The positive numbers on the right side reflect qualities that were more prevalent in the PKU, like some taxon from *Lachnospiraceae* and *Eggerthella lenta*. The gut volcano plot indicated that there were significant variations in the abundance of certain bacterial species between the two groups being studied (Figure 8).

A differential abundance analysis ALDEx2 showed no statistically significant differences in the oral microbiome between PKU and control.

## 4. Discussion

### 4.1. Analysis of Dietary Intake and Nutritional Requirements

A comparative analysis of the oral microbiome, gut microbiome, and dietary data was conducted involving two groups of children: those with PKU and a control group. The mean Phe level in the PKU group was 5.40 ± 2.90 mg/dL. Therefore, the studied children had a mild form of PKU (Phe level 120–360 μmol/L) [34]. Regular monitoring of Phe levels in the blood is crucial in the treatment of PKU, as it prevents the toxic effects of the accumulation of this amino acid [35]. Compared to previous research, our PKU and control groups took in more calories than previously reported patients (PKU = 1227.92 ± 187.9, control = 1277.56 ± 78.43) [7]. This may be attributed to age disparities between our children and another group (PKU = 4.24 ± 1.74; control = 6.06 ± 1.78) [7]. Our PKU group consumed less protein and more fat compared to the previous study of children [7] (PKU = 79.12 ± 15.41; control = 72.82 ± 15.21) (PKU = 16.12 ± 3.35; control = 37.75 ± 4.45), respectively. In our research, both groups had equal fibre consumption; however, the comparative analysis showed a difference from another study (PKU = 17.44 ± 3.42; control = 12.37 ± 0.97) [7]. The intake of digestible carbohydrates (g) was compared to previous research [7].

### 4.2. Impact of Therapeutic Diet and Nutritional Deficiencies in PKU

Comparing the nutrition of children with PKU with healthy children is crucial to understanding the differences in their dietary needs and the impact of diet on their development. Sufficient energy intake is crucial for individuals with PKU to maintain optimal levels of Phe in the bloodstream, especially in cases of classic PKU [36]. Fruits, some vegetables, and oils are the predominant products within the diet of these individuals. Children with PKU also consume food items that have a decreased Phe content, such as bread, pasta, rice, or cereals. Consuming items that have a high glycaemic index and are rich in fatty acids, particularly saturated ones, results in excessive energy intake, which may contribute to the issue of overweight and obesity [37]. Significant differences were observed across groups in the amount of plant protein intake. The PKU group consumed a lower amount of Phe due to the therapeutic diet, and this difference was statistically significant. Differences were seen in the intake of vitamins, including folates (statistically significant) and vitamins B12 and D, which were not statistically significant. The majority of these differences were due to a greater amount of animal-derived products, which are used as a protein source, in the control group. The results indicate that children with PKU often have deficiencies in certain vitamins, even while taking supplements. To summarise, the research revealed that there were no significant differences in total calorie consumption across the groups, although the energy sources varied between the two groups. Additionally, children with PKU have monotonous eating patterns compared to healthy children. Despite supplementation, children with PKU may be deficient in important nutrients that are involved in body growth.

### 4.3. Comparative Analysis of Gut Microbiota in PKU and Control Groups

Diverse studies have confirmed that nutrition has an impact on the composition of the gut microbiome. Studies on PKU patients suggest that diet may influence the interaction between the gut, microbiota, and brain. In this research, we analysed samples of stool and saliva taken from children with PKU and a control group without PKU. The sequencing was performed specifically for the V4 hypervariable region of the 16S rRNA gene. We analysed the gut microbiome and oral microbiome ecosystems, together with their diversity, in a group of individuals with PKU to investigate the impact of PKU on these microbiomes in contrast to a control group.

The comparative analysis of gut microbiota between PKU children and control groups revealed significant differences at various taxonomic levels, reflecting the complicated interaction between diet, disease, and microbial ecology. Our findings indicated that Firmicutes and Bacteroidetes were the predominant phyla in both groups but did not differ significantly (*p* = 0.71). Notably, only the phylum Euryarchaeota showed a significant increase in the control group (*p* = 0.01). Previous studies conducted on a group of children from Brazil showed that Bacteroidetes had a higher abundance compared to Firmicutes in both the PKU and control groups [7]. In a study of Uygur (China) children with PKU, the most prevalent phylum was Firmicutes (44.3%), followed by Actinobacteria (25%). In the healthy control group, more than 90% of the gut microbiome consisted of Firmicutes and Bacteroidetes, followed by minimal abundances of Actinobacteria and Proteobacteria [9]. Lower Bacteroidetes abundance was inversely linked with blood Phe levels in Chinese children. Bacteroides can use complex resistant glycans and maybe use other carbon sources by obtaining enzymes through heterologous gene transfer in the gut [38]. Even on a Phe-restricted diet, 27% of children had cognitive impairment and developmental delay. Among the various classes identified in this study, only two demonstrated statistical significance, Bacilli in PKU (*p* = 0.019) and Methanobacteria in controls (*p* = 0.01), which underscores potential functional implications. Bacilli release enzyme complexes (protease, amylase, cellulase, and lipase) that aid in the digestion of foods in the gastrointestinal tract (GIT) [39].

### 4.4. Comparison of PKU Microbiome in Different Populations

On the other hand, research conducted on children from the Italian population divided into two groups, PKU (with an average age of 10.0 ± 3.5) and mild hyperphenylalaninemia (MHP) (with an average age of 8.0 ± 3.4), revealed that children with PKU had a lower presence of *Faecalibacterium* spp. and a higher presence of *Blautia* spp. and *Clostridium* spp. (belonging to the *Lachnospiraceae* family) [8]. Our study compared the microbiomes of the PKU group and healthy controls, revealing a similar trend in the results, though they did not achieve statistical significance. In contrast, when comparing the microbiome of children with Brazilian PKU (age = 4.24 ± 1.74) to controls (age = 6.06 ± 1.78), it was observed that the PKU group had a reduced abundance of bacteria from the *Clostridiales* class, *Coprococcus*, *Lachnospira*, and *Ruminococcus* genera. A comparable trend was noted in our study (data not statistically significant). Moreover, there was a notable reduction in the number of other taxa within the PKU group from Brazil: the *Clostridiaceae*, *Erysipelotrichaceae*, and *Lachnospiraceae* families, as well as the *Dorea* and *Veillonella* genera. Our findings indicate a contrary trend, revealing an increase in the relative abundance of the aforementioned taxa within the PKU group, although this change was not statistically significant. Furthermore, their analyses showed an increase in the prevalence of *Prevotella* and *Akkermansia* in the PKU group, whereas in our results, this increase was only observed for *Akkermansia* [7]. The differing results observed in the conducted research compared to previous studies may be due to differences in patient age groups; sequencing platforms used; and regional, genetic, and dietary variations.

On the basis of our study, significant increases in *Eubacterium dolichum* and *Ruminococcus gnavus* in PKU (*p* = 0.015 and *p* = 0.044, respectively) and *Barnesiella intestinihominis*, *Coprococcus eutactus*, and *Faecalibacterium prausnitzii* in controls (*p* = 0.014, *p* = 0.01, and *p* = 0.014, respectively) further emphasise the nuanced microbial distinctions.

Additionally, this study investigated the composition within each group and sample. The samples in these two groups showed observable differences in the abundance of specific taxa, including *Ruminococcus gnavus*, *Shigella*, *Prevotella*, *Bacteroides*, *Bifidobacterium*, *Blautia*, *Faecalibacterium*, *Faecalibacterium prausnitzii*, *Prevotella copri*, *Rumminococcus*, and *Roseburia faecies*. These findings indicate that there were differences in the composition of gut bacteria between those with PKU and those without. These results highlight the impact of PKU and associated dietary restriction on the gut microbiota, which might have wider consequences for the metabolic and immunological well-being of these individuals. To understand the functional implications of these microbial differences and explore potential therapeutic interventions to modulate the gut microbiota for the benefit of PKU patients, further research is necessary. We identified *Streptococcus*, *Blautia*, *Lachnospiraceae*, *Oscillospora*, *Eggerthella lenta*, and *Faecalibacterium* at relatively high frequencies in the core members of samples from PKU. We identified those taxa even at reduced relative abundance criteria, indicating their abundance and frequency in the intestine of individuals with PKU. The presence of taxons with high detection thresholds and a diverse prevalence suggests that the gut microbiota composition is dynamic and individualised.

These findings suggest that PKU management and diet significantly shape gut microbiota composition, potentially influencing metabolic and immune functions, and highlight the need for further research to elucidate the clinical implications of these microbial shifts.

### 4.5. Influence of Nutrition and Short-Chain Fatty Acid (SCFA) Production in PKU

Our research and the reviewed literature reveal significant changes in the gut microbiota of children with PKU compared to controls, both intra-group and inter-group. The obtained microbiome differences may be modulated by the plasma Phe concentration. Compared to the control group, the PKU group contained a limited number of microbes involved in starch and sucrose metabolism; glycolysis/gluconeogenesis; and in the biosynthesis of the amino acids Phe, Tyr, and tryptophan (Trp) [7].

Bacteroidetes and Firmicutes are the two most common types in the human gut. Butyrate is produced by Firmicutes, whereas acetate and propionate are produced by Bacteroidetes [40]. Short-chain fatty acids such as acetate, propionate, and butyrate provide antioxidant activity. Acetate functions as a substrate for lipogenesis and gluconeogenesis, whereas butyrate and propionate may modulate immunological and intestinal physiology [41]. These substrates ferment into short-chain fatty acids in anaerobic environments, which host cells can use as a source of ATP [42]. This group of metabolites also interact with the immunological system of the host [43]. Firmicutes abundance and the Firmicutes/Bacteroidetes ratio increased significantly in obese individuals. Our findings indicate that the F/B ratio in the gut microbiome was 1.55 for individuals with PKU compared to 1.39 for the control group. Analyses of the gut microbiota of obese and lean individuals revealed a significant reduction in the abundance of Bacteroidetes in the obese group as compared to the lean group [44]. Researchers have linked Firmicutes abundance and the Firmicutes/Bacteroidetes ratio to the digestion of some indigestible polysaccharides, the subsequent production of monosaccharides and SCFAs such as acetate and butyrate, and the extraction of energy from compounds that faeces would otherwise diminish [45]. When compared to previous studies for PKU children [8,46], the Firmicutes type showed an increased relative abundance of *Blautia* and *Clostridium* in the gut microflora of PKU patients, which was confirmed in our study in the case of *Blautia*. A high-fibre diet and supplementation with PKU low-protein products containing inulin may influence the abundance of this genus [46]. It was observed that *Blautia* induces the cytokine tumour necrosis factor-alpha (TNF-alpha), a component of the acute immunological phase, to be secreted [47].

### 4.6. Impact of Diet on Microbiome Composition in Children with Phenylketonuria

Increased carbohydrate consumption in low-Phe diets enhances the glycaemic index (GI) and glycaemic load (GL), resulting in a varied substrate quality for microbial fermentation [46]. In our investigation, GI was marginally elevated in the PKU group (data not statistically significant). In a past study, researchers found increased daily GI in PKU children. It was been shown that *Faecalibacterium prausnitzii*, unclassified *Ruminococcaceae*, and *Roseburia* spp. correlated negatively with GI, but unclassified *Lachnospiraceae* correlated positively. Based on indicator species, *F. prausnitzii* was linked to MHP and *Ruminococcus bromii* to PKU [8]. Our results revealed a similar tendency; however, the statistically significant data pertained only to *F. prausnitzii* (*p* = 0.0140) and *Ruminococcaceae* (*p* = 0.0190). In PKU patients, overall SCFAs and butyrate production decreased, as did the depletion of butyrate-producing F. *prausnitzii* and *Roseburia* spp. and lactate-producing *Lactobacillus* spp. Both the quality and quantity of carbohydrate intake are involved in determining the observed changes in the Firmicutes population in the PKU patients’ microbiota [8]. Modified diets used to lower Phe intake in PKU patients may cause an imbalance in the Firmicutes/Bacteroidetes ratio, which is connected to higher body mass index (BMI) in children and, further, to childhood obesity. The microbiota of PKU patients can undergo substantial changes, shifting towards a microbial composition possibly favouring less effective glycaemic control, increased insulin resistance, and weight gain when combined with chronically reduced AA intake, often inadequately compensated by protein substitutes [10].

Previous studies indicate a decrease in *Faecalibacterium* in samples from both adults and children with PKU [7,8,48]. According to other research, the microbiome of people with PKU is deficient in two taxa, *Faecalibacterium* and *Roseburia*. Our data demonstrated a comparable trend, although they did not demonstrate statistical significance. The concentration of total SCFA in faeces and butyrate is reduced as a result [46,49]. The bacteria of the genus *Faecalibacterium* mainly consume dietary fibre by metabolising it into SCFAs, e.g., butyrate, which, together with microbial anti-inflammatory molecules (MAM), gives these bacteria anti-inflammatory properties [50,51]. *Faecalibacterium prausnitzii* has been one of the primary butyrate producers in healthy people and usually comprises more than five of the total gut bacterial population [52], the same results we had. Patients with colitis, inflammatory bowel disease (IBD), and Crohn’s disease have been observed to have decreased concentrations of the *Faecalibacterium* in stool samples [53,54]. In the case of our PKU patients, it was observed that their diet contains statistically considerably less protein than the control, although there is no statistically significant difference in the amount of fibres.

The abundance of *Lachnospiraceae* was slightly higher in PKU than in control, and this genus was more prevalent in PKU patients’ microbiomes than in healthy controls [55], who reported a reduced prevalence of *Lachnospiraceae* [7,48]. *Lachnospiraceae* members belong to the major producers of SCFAs, mainly propionate, which benefit the host’s health. On the other hand, different taxa of the genus are linked to several diseases, including metabolic syndrome, obesity, diabetes, liver disease, inflammatory bowel disease, chronic kidney disease, depression, and multiple sclerosis [56]. Researchers emphasised the necessity for more study on *Lachnospiraceae* regulation to prevent and treat the aforementioned disorders because the literature’s data on this family are conflicting [56].

It is worth noting that there are several taxa whose presence and frequency of occurrence in the study samples are significant, although comparisons of PKU samples and controls did not show statistically significant differences in gut microbiome composition. One genus present in all study samples is *Prevotella* spp., whose abundance was twice as high in samples from healthy children as in samples collected from children with PKU. The presence of *Prevotella* spp. (e.g., *P. copri*) has been characterised well in the gut microbiome, and its relative abundance was inversely correlated with Bacteroides. Our research revealed a statistically significant lower protein consumption in PKU patients on a Phe-free diet, which is likely connected to the result for Bacteroides [57]. *Prevotella* is typically linked to increased fibre consumption [58]. The *P. copri* complex was reported in the context of non-Western diet patterns high in carbohydrates, resistant starch, and fibre. A *Prevotella*-rich gut microbiome promotes weight loss and cholesterol reduction in fibre-rich diet participants, optimises glucose metabolism, and increases succinate metabolism. However, further research has shown that *P. copri* is favourable to glucose homeostasis and the host metabolism [59,60,61]. *Prevotella* spp. have been associated with a variety of disorders, including inflammatory disorders, viral infections, and bacterial vaginosis. It is unknown, however, how they contribute to these disorders [59]. *Prevotella* contains a range of polysaccharide-digesting enzymes that are critical for maintaining digestion dynamics and intestinal homeostasis. The greater the richness of *Prevotella* spp., the greater the fermentability of the microbiome benefits the human gut [59]. More study is needed since *P. copri* has multiple subspecies strains that alter the host phenotype in different ways.

### 4.7. Differences in Alpha and Beta Diversity Metrics in PKU and Control Gut Microbiome

Our results indicated that the gut microbiota of PKU patients had lower alpha diversity in comparison to the control group, as shown by lower Observed, Shannon, and Simpson values. The decreased diversity in the gut microbiota in PKU patients may be due to dietary restrictions or metabolic disorders linked to the disease. The analysis of the gut microbiota revealed a reduction in Biodiversity Indicators, namely, alpha diversity (Shannon, Simpson, and Observed), in the PKU group, consistent with findings from previous research [3,7,9]. The PERMANOVA analysis conducted on the gut microbiome showed the significant effects of the treatment groups (PKU and control) on the diversity of microbial community complexion. Furthermore, a statistical test examining the dispersion of the microbial population among treatment groups found a significant change. Further pairwise comparisons provided additional information supporting a significant difference in microbial composition between the control and PKU groups. On the beta diversity substrate, there are statistically significant variations in gut groups [3,8,9].

The preceding discussion compares our findings with those of other researchers. Alterations in outcomes may be caused by antibiotic consumption, microbiome differences between groups (PKU and control), individual variability, dietary influences, and the manifestations of phenylketonuria disease itself [7].

### 4.8. Comparative Analysis of Oral Microbiota in PKU and Control Groups

The salivary microbiome is influenced by a variety of factors, including age, diet, oral cavity condition, disorders such as edentulism or caries, chronic diseases, and medications. Diet, disease, and age significantly determine the diversity of the intestinal microbiota [62]. Research conducted on PKU patients and controls in the German population, ranging from 6 to 68 years old, showed no significant differences between the study groups in the abundance of *Neisseria* and *Prevotella* [63]. Furthermore, *Haemophilus* was shown to be the most frequently occurring taxon in those with PKU [63]. While *Streptococcus* was more prevalent in PKU compared to the control group, the findings did not show statistical significance [63]. The outcomes of our study indicate that *Streptococcus* and *Neisseria* in PKU (*p* = 0.0079 and 0.016, respectively) were the most prevalent taxon in children with PKU. This result is consistent with previous research on the oral microbiome. Different research indicates that *Streptococcus* constitutes a normal component of the oral microbiome, while other studies show that they have been linked to dental caries and other oral diseases [64,65,66]. *Streptococcus mutans* and *Streptococcus sobrinus* were recognised as the primary agents responsible for producing dental caries. *Streptococcus mutans* is the predominant bacterial species found in carious lesions, making it the primary causative agent of dental caries [65]. *Streptococcus infantis* (*p* = 0.032) and *Prevotella nanceiensis* (*p* = 0.032) were more common in PKU and controls, respectively, in our results. *Streptococcus infantis* is associated with oral health [67], indicating that this commensal species was significantly negatively correlated with the growth rate of various pathogens, including *Treponema medium*, suggesting that this commensal microorganism may play an important role in colonisation resistance [68]. *Prevotella nanceiensis* exhibited higher prevalence in children diagnosed with HSP (Henoch–Schönlein Purpura) and had a positive correlation with the levels of IgA [69].

The oral microbiome of PKU patients from the Latvian population shows greater caries activity and periodontal disease due to their carbohydrate-rich diet. Plaque, which all PKU patients have, is a key risk factor for periodontal disease [64]. The control group had considerably more *Alloprevotella* than the PKU group. *Actinomyces*, *Capnocytophaga*, and *Haemophilus* were more abundant in PKU patients who rigorously followed the diet than in the control. Patients with phenylketonuria who only partly followed the diet showed an increase in the presence of *Capnocytophaga*. In comparison to the controls, the PKU group we examined exhibited decreased levels of the aforementioned genera; however, these findings were not statistically significant. Additionally, both PKU patients who adhered to the diet and those who did not showed an increase in the presence of *Porphyromonas* [64]; similar results were obtained for our PKU cohort. Recent investigations have linked *Actinomyces* to purulent and granulomatous inflammatory lesions and *Capnocytophaga* and *Porphyromonas* to periodontal disease [70,71]. Due to the fact that our research did not involve dental exams, we are unable to determine whether or not individuals in either the PKU or control group had any dental disease.

We found differences in the oral microbiomes of children with PKU and healthy controls in taxa, including *Streptococcus*, *V. parvula*, *P. melaninogenica*, *P. pallens*, *Rothia*, and *Porphyromonas*. *Streptococcaceae*, *Neisseriaceae*, and Gemellacea were dominant in PKU, while *Veillonella* and *Actinomyces* were moderately common.

The obligatory anaerobic *Oribacterium*, which is a member of the *Lachnospiraceae* family, was identified in lower amounts in our PKU group than in the control group in the oral microbiome. The characterisation of *Oribacterium* showed that the metabolic end product was acetate [72].

The previously indicated changes in the diet and microbiome of children and adults with PKU result from the fact that parents watch over children’s diets and prepare meals. Adults are less strict about maintaining a restrictive diet, allowing themselves to deviate. Some patients discontinue their diet altogether but come back to it when disturbing symptoms appear [73].

### 4.9. Alpha and Beta Diversity Within Oral Microbiota

No significant differences in oral alpha and beta microbial diversity were seen between the control and PKU-treated groups, as shown by similar results for Observed, Shannon, and Simpson indexes; the same results were reported in previous research [63]. The results indicate that oral PKU treatment had no significant impact on the total microbial diversity as compared to the control group. The PERMANOVA analysis conducted on the oral microbiota demonstrated that there was no statistically significant difference in dispersion (variance) between the control and PKU groups. Therefore, the significant outcome of the PERMANOVA study may be mostly attributable to variations in the central tendency (location) of community composition rather than variations in dispersion. The pairwise comparisons between the control and PKU groups did not demonstrate any significant differences, as shown by the high *p*-values. This implies that the overall importance of the PERMANOVA study was affected by minor variations that did not have individual significance.

### 4.10. Clinical Implications for Dietary Management and Monitoring

The results of the comparative investigation between children with PKU and healthy controls reveal critical disparities in food intake, nutritional deficits, and microbial composition, highlighting important implications for clinical treatment and health policy. The distinct dietary needs of children with PKU and the effects of their specialised low-protein diets on metabolic health and microbiota composition indicate the need for a comprehensive strategy that transcends the mere regulation of phenylalanine (Phe) levels.

This research underscores the significance of nutritional balance in treating PKU, especially in maintaining sufficient amounts of critical vitamins and minerals. Despite supplementation, children with PKU often exhibit shortages in essential nutrients, such as folates, and variations in microbiota composition, which may affect long-term metabolic health. Recent scientific and medical advancements indicate that dietary approaches are not universally applicable, as individual responses to dietary interventions differ markedly, significantly influencing the microbiome [74]. Environmental factors, including dietary exposure, significantly influence the composition of the microbiome. The influence of the microbiome on human physiology is well established, affecting digestion, nutrient absorption, mucosal immune response, and synthesis [75]. Research with a larger cohort of patients, including the metatranscriptome and metabolome, is necessary. Future outcomes may assist in delineating a panel of the requisite functional microbiota. Clinicians might include regular, thorough nutritional evaluations and microbiota testing in standard PKU management. Currently, continuous monitoring should prioritise regulating Phe and Tyr levels while optimising food intake and supplements to facilitate proper organismal growth and function. This is especially significant given discoveries that suggest a heightened risk of obesity and other metabolic or neurological issues associated with dietary composition.

### 4.11. Implications for Microbiome-Targeted Therapies

The notable microbiome differences between the PKU and control groups suggest the possibility of microbiome-targeted treatments. The identified disparities in beneficial bacterial populations, including reduced levels of *Faecalibacterium* and *Roseburia* in PKU patients, indicate that probiotic or prebiotic interventions may be advantageous for microbiome health management. Customising therapies to promote the proliferation of these taxa may alleviate inflammation and enhance gastrointestinal health. The presence of certain bacteria, such as *Streptococcus* and Lactobacillales, in increased quantities among PKU patients may indicate particular microbial targets for improving health outcomes in PKU care. The application of probiotics or prebiotics necessitates a deeper comprehension of the relationship between gut microbiota and nutritional therapy [76]. Data indicate that the microbiome may be influenced positively or negatively by nutritional and pharmacological interventions in patients with PKU, as well as other inborn errors of metabolism (IEM) [74,77,78]. It is essential to examine the metabolic function of gut microbiota in patients with PKU. This may enhance intervention strategies by facilitating beneficial pathways and enabling personalised biotherapeutics.

### 4.12. Health Policy Implications: Support for Nutritional and Microbiome Research in PKU

Diet therapy remains the primary treatment for PKU, encompassing three components: restriction of natural protein intake, consumption of low-protein foods, and supplementation with phenylalanine-free amino acids [6]. The distinct and intricate nutritional requirements of individuals with PKU underscore the necessity for policies that guarantee access to specialised dietary products and ongoing nutritional monitoring. Furthermore, policies that promote research on the microbiome and dietary interventions in PKU may enhance the development of personalised treatment options, potentially reducing the risk of secondary health complications related to PKU, including obesity, immune dysregulation, and developmental challenges [36].

Future research should investigate strategies for microbiome modulation and nutrient optimisation in PKU diets, thereby expanding the therapeutic options for PKU management and supporting health policy initiatives focused on enhancing long-term care for these patients.

## 5. Conclusions: Impact of PKU and Dietary Management on Microbiota Composition

Based on the analysis of the diet, it can be concluded that the diet of people with PKU differs significantly from that of the controls. Therefore, different substrates are provided for the bacteria living in the oral cavity and in the intestine. Earlier studies indicated that the intestinal microbiome of people with PKU differs from the control group. Multiple research studies have substantiated that nutrition exerts an influence on the composition of the gut microbiota. Investigations on individuals with PKU indicate that nutrition can impact the interplay of the gastrointestinal tract, microbiome, and brain. A comprehensive investigation including a larger cohort of PKU patients and healthy individuals is necessary. In order to obtain accurate conclusions and validate the findings of this study, it is imperative to increase the sample size and acquire comprehensive nutrition-related data. In-depth analyses of the microbiome, including the mycobiome and virome, as well as advanced metatranscriptomic and metabolomic analyses, would allow for the determination of the relationships between the composition and functioning of the microbiota associated with dietary changes (including probiotics, prebiotics, or medications) in healthy individuals and those with phenylketonuria.

## Figures and Tables

**Table 1:** Nutritional characteristics of the PKU and control groups.

	Control		PKU Patient		*p*-Value
	Average	SD	Average	SD	
Energy (kcal)	1494	262	1684	748	0.6968
Total proteins (g)	61.43	17.88	41.28	5.83	0.2159
Plant proteins (g)	17.03	3.22	4.28	3.07	0.0013 *
Animal proteins (g)	35.30	16.43	4.28	6.55	0.0688
Fat (g)	43.43	12.01	61.00	32.15	0.4147
MUFA (g)	17.70	3.38	16.18	7.41	0.7576
PUFA(g)	7.93	2.68	12.58	15.89	0.5959
*n*-3	0.93	0.55	0.78	1.03	0.8213
*n*-6	3.40	2.31	2.60	2.20	0.6599
SFA (g)	16.83	3.16	19.48	4.27	0.4117
Cholesterol (mg)	209.70	176.34	74.33	66.78	0.2159
Digestible carbohydrates (g)	206.07	27.66	208.10	106.16	0.9760
Fibre (g)	22.67	2.88	20.25	7.54	0.6271
Starch (g)	79.00	31.83	75.10	87.01	0.5959
Phenylalanine	2608.70	720.98	739.83	445.61	0.0079 *
Glycaemic index	32	11	36	15	0.7280
Sodium (mg)	1483.60	450.22	1092.83	1082.39	0.8597
Potassium (mg)	2438.85	1159.52	3515.57	1541.71	0.3362
Calcium (mg)	189.63	101.41	664.73	378.10	0.0564
Magnesium (mg)	181.58	42.75	275.33	99.92	0.2159
Iron (mg)	6.43	2.33	10.97	3.35	0.0856
Copper (mg)	0.58	0.29	0.90	0.44	0.5925
Zinc (mg)	3.03	1.32	7.93	3.20	0.0518
Vitamin B1 (mg)	1.03	0.67	0.50	0.38	0.5925
Vitamin B2 (mg)	1.27	0.76	0.40	0.16	0.0718
Vitamin B3 (mg)	15.13	4.31	7.68	6.47	0.1476
Folates (µg)	387.87	66.16	153.65	51.70	0.0013 *
Vitamin B12 (µg)	2.30	1.35	0.40	0.42	0.0518
Vitamin B6 (mg)	2.07	1.17	1.18	0.68	0.2060
Vitamin A (µg)	2495.93	2908.79	371.45	243.91	0.5959
Vitamin C (mg)	164.37	95.66	92.80	54.89	0.2609
Vitamin D (µg)	1.77	1.19	0.33	0.28	0.0518
Vitamin E (mg)	10.03	8.53	6.23	2.50	0.7188
Β-carotene (µg)	19,673.77	26,976.52	1859.60	1469.60	0.2159

Statistical significance between groups at *p* < 0.05 was marked with ‘*’. MUFA—monounsaturated fatty acids; PUFA—polyunsaturated fatty acids; *n*-3—omega-3 fatty acids; *n*-6—omega-6 fatty acids; SFA—saturated fatty acids.

## Data Availability

The original 16S sequencing raw data presented in the study are openly available in NCBI SRA for gut microbiome under the accession number PRJNA1161376 and oral microbiome under accession number PRJNA1161359.

## Supplementary Materials

The following supporting information can be downloaded at: https://www.mdpi.com/article/10.3390/nu16223915/s1, Figure S1: Core microbial taxa in PKU gut samples and their relative abundance; Figure S2: Core microbial taxa in oral PKU samples and their relative abundance; Table S1: Gut microbial phylum abundance and statistical comparison between PKU and control groups; Table S2: Gut microbial class abundance and statistical comparison between PKU and control groups; Table S3: Gut microbial order abundance and statistical comparison between PKU and control groups; Table S4: Gut microbial family abundance and statistical comparison between PKU and control groups; Table S5: Gut microbial genus abundance and statistical comparison between PKU and control groups; Table S6: Gut microbial species abundance and statistical comparison between PKU and control groups; Table S7: Alpha and beta diversity metrics of gut microbiota in PKU and control groups, with PERMANOVA analysis; Table S8: Oral microbial phylum abundance and statistical comparison between PKU and control groups; Table S9: Oral microbial class abundance and statistical comparison between PKU and control groups; Table S10: Oral microbial order abundance and statistical comparison between PKU and control groups; Table S11: Oral microbial family abundance and statistical comparison between PKU and control groups; Table S12: Oral microbial genus abundance and statistical comparison between PKU and control groups; Table S13: Oral microbial species abundance and statistical comparison between PKU and control groups; Table S14: Alpha and beta diversity metrics of oral microbiota in PKU and control groups, with PERMANOVA analysis.
