# Supplementary material for: Changes in the Gut and Oral Microbiome in Children with Phenylketonuria in the Context of Dietary Restrictions—A Preliminary Study
Source: Nutrients. 2024 Nov 16;16(22):3915. doi: 10.3390/nu16223915 (PMC11597790; doi:10.3390/nu16223915)
Supplement: Supplementary file 1 [file nutrients-16-03915-s001.zip › Supplementary materials - Figures S1 and S2.pdf]

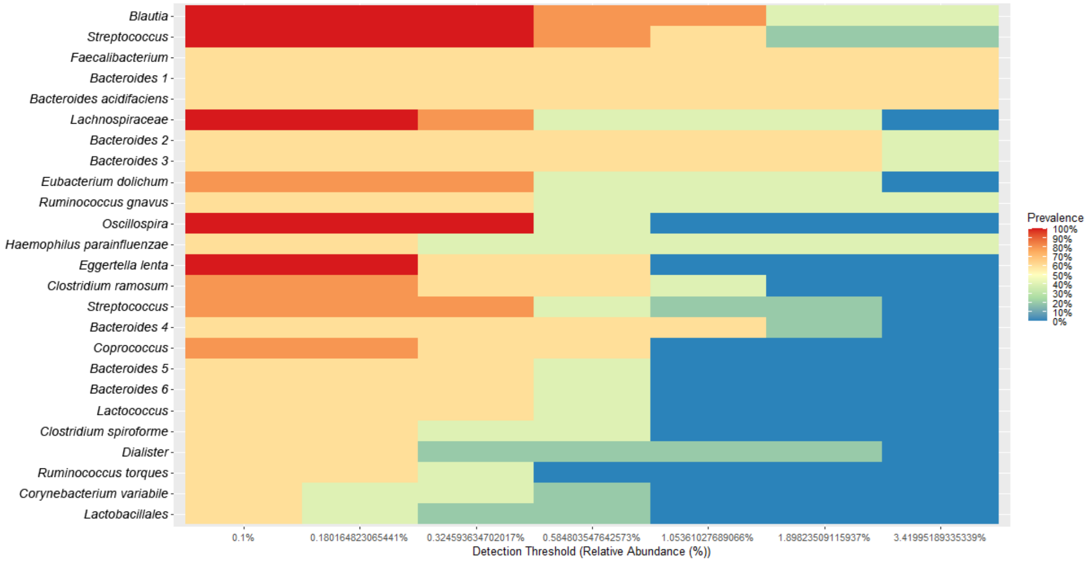


**Figure S1.** Core microbial taxa in PKU gut samples and their relative abundance. Heatmap showing the prevalence of different microbial taxa in gut samples from individuals with PKU across varying detection thresholds (relative abundance). The y-axis lists the taxa, and the x-axis represents the detection threshold in percentage of relative abundance. The color gradient from red to blue indicates the prevalence of each taxon, with red representing 100% prevalence and blue representing 0% prevalence.

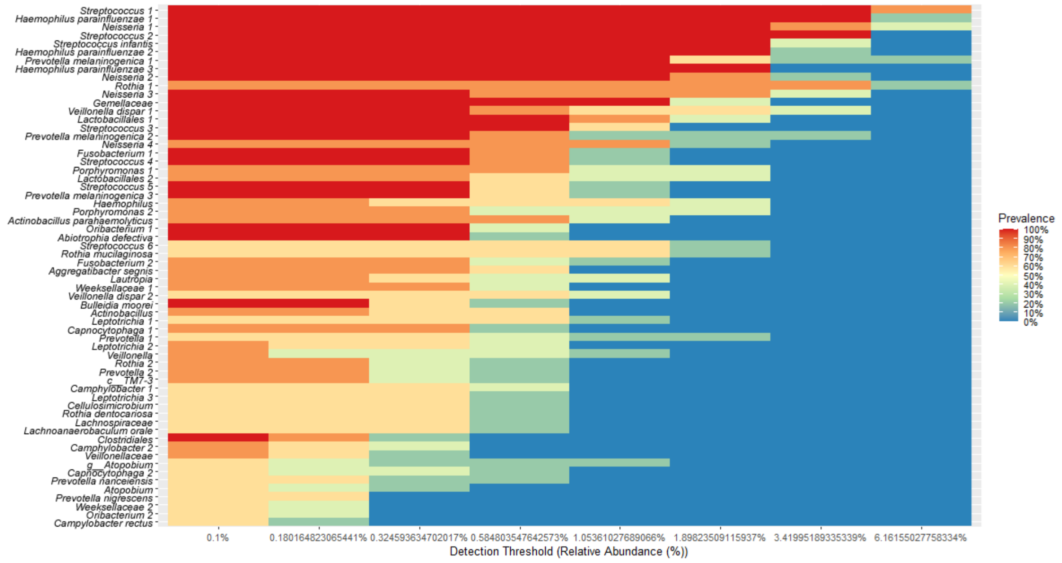


**Figure S2.** Core microbial taxa in oral PKU samples and their relative abundance. Heatmap showing the prevalence of different microbial taxa in oral samples from individuals with PKU across varying detection thresholds (relative abundance). The y-axis lists the taxa, and the x-axis represents the detection threshold in percentage of relative abundance. The color gradient from red to blue indicates the prevalence of each taxon, with red representing 100% prevalence and blue representing 0% prevalence.
